# Attentional state-synchronous peripheral electrical stimulation during action observation induced distinct modulation of corticospinal plasticity after stroke

**DOI:** 10.3389/fnins.2024.1373589

**Published:** 2024-03-28

**Authors:** Chang Hyeon Jeong, Hyunmi Lim, Jiye Lee, Hye Sun Lee, Jeonghun Ku, Youn Joo Kang

**Affiliations:** ^1^Department of Rehabilitation Medicine, Nowon Eulji Medical Center, Eulji University, Seoul, Republic of Korea; ^2^Department of Biomedical Engineering, Keimyung University, Daegu, Republic of Korea; ^3^Biostatistics Collaboration Unit, Yonsei University College of Medicine, Seoul, Republic of Korea

**Keywords:** brain computer interface, electrical stimulation therapy, action observation, cortical synchronization, cortical excitability, rehabilitation, stroke

## Abstract

**Introduction:**

Brain computer interface-based action observation (BCI-AO) is a promising technique in detecting the user's cortical state of visual attention and providing feedback to assist rehabilitation. Peripheral nerve electrical stimulation (PES) is a conventional method used to enhance outcomes in upper extremity function by increasing activation in the motor cortex. In this study, we examined the effects of different pairings of peripheral nerve electrical stimulation (PES) during BCI-AO tasks and their impact on corticospinal plasticity.

**Materials and methods:**

Our innovative BCI-AO interventions decoded user's attentive watching during task completion. This process involved providing rewarding visual cues while simultaneously activating afferent pathways through PES. Fifteen stroke patients were included in the analysis. All patients underwent a 15 min BCI-AO program under four different experimental conditions: BCI-AO without PES, BCI-AO with continuous PES, BCI-AO with triggered PES, and BCI-AO with reverse PES application. PES was applied at the ulnar nerve of the wrist at an intensity equivalent to 120% of the sensory threshold and a frequency of 50 Hz. The experiment was conducted randomly at least 3 days apart. To assess corticospinal and peripheral nerve excitability, we compared pre and post-task (post 0, post 20 min) parameters of motor evoked potential and F waves under the four conditions in the muscle of the affected hand.

**Results:**

The findings indicated that corticospinal excitability in the affected hemisphere was higher when PES was synchronously applied with AO training, using BCI during a state of attentive watching. In contrast, there was no effect on corticospinal activation when PES was applied continuously or in the reverse manner. This paradigm promoted corticospinal plasticity for up to 20 min after task completion. Importantly, the effect was more evident in patients over 65 years of age.

**Conclusion:**

The results showed that task-driven corticospinal plasticity was higher when PES was applied synchronously with a highly attentive brain state during the action observation task, compared to continuous or asynchronous application. This study provides insight into how optimized BCI technologies dependent on brain state used in conjunction with other rehabilitation training could enhance treatment-induced neural plasticity.

## 1 Introduction

There are various sequelae of stroke, and disability of upper extremity motor function is one of the most common and persistent (Lai et al., 2002; Langhorne et al., 2011). Neuroplasticity is a term that describes the ability of the brain to create new neural connections, acquire additional functions, and adapt to compensate for neural damage (Murphy and Corbett, 2009). These processes play a crucial role in the recovery of upper extremity motor function after stroke. It is important to emphasize the significance of research that focuses on rehabilitation strategies to enhance brain plasticity. Various neuromodulation techniques have been clinically employed to enhance motor recovery by promoting neuroplasticity. These include non-invasive brain stimulation, neuromuscular electrical stimulation, paired associative stimulation, and application of the brain-computer interface (BCI) technique (Ting et al., 2021). However, individual treatment strategies do not induce sufficient neural plasticity for motor recovery. Consequently, various neurotechnologies have recently been incorporated into conventional rehabilitation techniques to improve their overall efficacy. The effectiveness of therapies for motor recovery can be enhanced by combining protocols based on various mechanisms, rather than utilizing each treatment individually. This approach promotes a more stable and synergistic motor recovery (Takeuchi and Izumi, 2015).

Rehabilitation strategies for patients with a stroke often integrate action observation (AO) based on the theory of mirror neuron system (MNS) activation to enhance motor cortical plasticity and improve upper extremity function (Franceschini et al., 2012; Tani et al., 2018; Mancuso et al., 2021). AO stimulates neural plasticity by engaging the MNS, which responds when individuals perform specific actions and observe the same motor actions (Fadiga et al., 1995, Rizzolatti et al., 2021). This paradigm has the advantage of being applicable even to severely paralyzed stroke patients, because it is not dependent on motor activity. However, in the conventional AO paradigm, it is difficult for patients affected by stroke to maintain prolonged attention during training session without feedback or rewards. Thus, it is difficult to estimate whether patients actively engage in AO training.

Brain-computer interfaces (BCI) have been developed to evaluate cortical potential and offer brain state-dependent feedback to aid in rehabilitation (Ting et al., 2021). The BCI technology has great potential in the field of neurorehabilitation, and can be applied alone or in combination with traditional rehabilitation techniques. As an example, a BCI-based AO game that provides real-time feedback to patients regarding their attention to a blinking action video through steady-state visual -evoked potentials (SSVEPs) was introduced (Lim and Ku, 2017). This strategy elicited greater MNS activation compared with the AO paradigm applied alone in the unaffected and affected hemispheres of patients with a history of chronic stroke (Choi et al., 2019).

Peripheral electrical stimulation (PES) promotes brain plasticity by generating afferents and increasing corticospinal excitability (Pascual-Leone et al., 1995, Everaert et al., 2010; Veldman et al., 2018). This process is associated with motor learning and hastens motor recovery in stroke patients (Stefan et al., 2000, Ward, 2005; Di Pino et al., 2014). PES is a widely used rehabilitation technique after stroke because it is easily accessible and applicable to severely paralyzed patients. The motor learning effects of AO are transient and need to be complemented by motor training to maintain and maximize their impact (Zhang et al., 2011; Larssen et al., 2021). In another study, the combined AO and PES paradigm induced corticospinal facilitation similar to that achieved through real motor training, resulting in enduring changes in brain plasticity (Bisio et al., 2015, 2017).

A previous study introduced the concept of attentional state-dependent PES during the AO paradigm using the BCI-SSVEPs protocol (Kim et al., 2022). The results indicated that the attentional state-dependent PES task was superior to AO alone or to a simple combination of AO and PES in facilitating corticospinal plasticity in both patients with stroke and in healthy individuals (Kim et al., 2022). Moreover, this paradigm is effective in enhancing sensorimotor cortical activation in the affected hemispheres of stroke patients (Lim et al., 2023). BCI interventions decode the user's attentive observation during the AO task, provide rewarding visual feedback and activate afferent pathways via electrical stimulation. Closed-loop stimulation paradigms of this nature seek to induce more synchronized patterns of neuronal activity enhancing Hebbian plasticity (Hebb, 1949).

However, it has been consistently demonstrated that simple combinations of various neurotechnologies do not always exhibit synergy in stroke rehabilitation (Takeuchi and Izumi, 2015; Coscia et al., 2019). From this perspective, how different PES pairings during BCI-AO tasks differentially impact corticospinal plasticity has not been fully explored in previous studies. Hence, the next step requires an in-depth exploration of the different pairings of PES during BCI-AO training that influence corticospinal neuroplasticity.

In this study, we explored four combinations of PES application during BCI-AO tasks (absence of PES, and continuous, synchronous, and asynchronous application). Consequently, our experiment aimed to assess how synchronous stimulation, aligned with an individual's attentional state in the BCI-AO paradigm and combined with PES, affects corticospinal activation and neural plasticity after stroke. This investigation involved analyzing corticospinal excitability via transcranial magnetic stimulation (TMS) and quantifying mu suppression in electroencephalographic (EEG) activity.

## 2 Materials and methods

### 2.1 Participants

Patients enrolled in the study included (1) patients with mild to moderate upper extremity hemiplegia among patients with the first stroke diagnosed by radiological examination, (2) patients with chronic stroke at least 6 months after onset, (3) medically stable patients, and (4) patients with a stroke who were able to respond appropriately to verbal instructions. Patients were excluded if they had (1) contraindications for TMS, such as intracranial metal, pacemaker, or implant insertion; (2) mental disorders, such as depression and apraxia; (3) visual impairment or inability to communicate, (4) a history of seizures, and (5) absence of an MEP (motor evoked potential) response in the affected first dorsal interosseous (FDI) muscle. The Institutional Review Board of the university-affiliated hospitals approved the study protocol (EMCS 2022-07-012-002), and all participants provided written informed consent. Detailed clinical data of the patients with stroke and data on age and sex are provided in Table 1.

**Table 1:** Clinical and demographic characteristics of the stroke patients included in the study.

**Patients number**	**Sex**	**Age**	**Onset**	**Etiology**	**Site of lesion**	**mRS**	**FMA-UE**	**MMSE**	**MBI**	**MAS**
1	F	55	2Y9M	Hemorrhage	Rt. BG (Subcortex)	3	28	28	100	G1
2	M	50	2Y4M	Hemorrhage	Lt. Thalamus (Subcortex)	1	64	28	89	G0
3	M	59	9Y	Infarction	Rt. MCA (Cortex & Subcortex)	1	64	29	100	G0
4	M	58	1Y	Hemorrhage	Rt. Thalamus (Subcortex)	1	60	27	92	G0
5	M	65	7Y	Hemorrhage	Lt. BG (Subcortex)	1	60	29	97	G0
6	M	64	6M	Hemorrhage	Rt. BG (Subcortex)	2	62	24	53	G0
7	F	70	1Y1M	Infarction	Rt. BG (Subcortex)	1	64	29	97	G0
8	M	67	10M	Hemorrhage	Lt. Thalamus (Subcortex)	2	66	26	97	G0
9	F	59	9M	Hemorrhage	Lt. Thalamus (Subcortex)	2	62	29	93	G1
10	M	65	1Y1M	Infarction	Lt. SC (Subcortex)	2	64	29	95	G0
11	M	79	9M	Infarction	Rt. MCA (Cortex & Subcortex)	1	64	29	98	G0
12	F	62	6M	Infarction	Lt. BG (Subcortex)	1	64	29	97	G0
13	M	68	6M	Hemorrhage	Rt. CR (Subcortex)	1	64	29	97	G0
14	M	73	7M	Infarction	Lt. SC (Subcortex)	1	63	29	97	G0
15	F	65	11M	Hemorrhage	Rt. BG (Subcortex)	3	39	29	91	G0

Rt, Right; Lt, Left; mRS, Modified Rankin Scale; EHI, Edinburgh Handedness Inventory; FMA-UE, Fugl-Meyer Assessment For Upper Extremity; MMSE, Mini-mental State Examination; MBI, Modified Barthel Index; MAS, Modified Ashworth Scale; BG, Basal ganglia; IC, Internal capsule; CR, Corona radiata; MCA, Middle cerebral artery; SC, Striatocapsular.

### 2.2 Experimental setup

**Figure 1:**
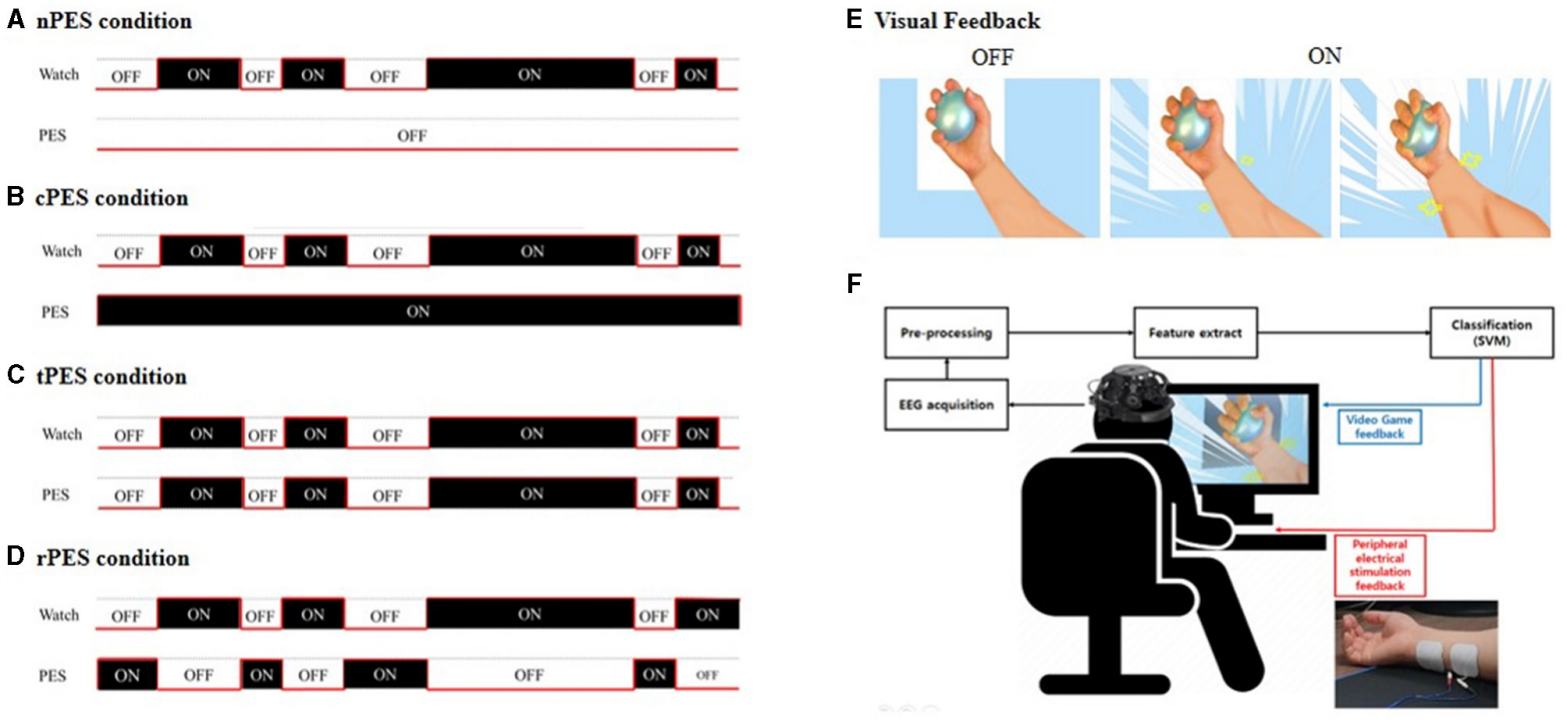
In the BCI-AO without PES application **(A)**, patients received visual feedback whether their attentive watching was assessed by detector without the application of PES. The BCI-AO with continuous PES application **(B)**, refers to a condition in which continuous PES was applied while the video played on the screen irrespective of whether patient's attentive watching was verified or not. In the BCI-AO with triggered PES application **(C)**, PES was turned on and off synchronously depending on whether the patient performed attentive watching or not. In the BCI-AO with reverse PES application **(D)**, PES application was operated in the opposite manner compared to the tPES condition. **(E)** Patients viewed a video with repetitive grasping actions. **(F)** The AO clip, modulated at 15 Hz, facilitated classifier identification of steady-state visually evoked potential components from EEG. The BCI-AO program was designed to provide rewarding visual feedback, highlighting the enlargement of muscles in the hand and forearm based on the level of attentive watching shown by the patient. AO, action observation; PES, peripheral electrical stimulation; BCI-AO, brain-computer interface-action observation; SVM, support vector machine; EEG, electroencephalogram.

**Figure 2:**
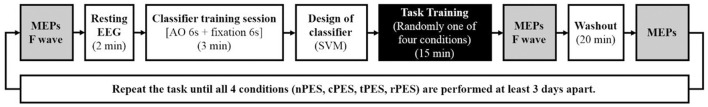
Experimental procedure and time points for measuring the latency and amplitude of MEPs and F wave latency. MEP parameters were assessed at three intervals: baseline (Pre), immediately after the task training (Post 0), and 20 min after completing each task (Post 20). The four tasks were conducted in a randomized order with intervals of at least 3-days.

We developed a video game using the BCI-AO paradigm, offering real-time visual cue feedback through the BCI. The patient's level of attentive watching was assessed by measuring EEG intensities in the beta and mu bands as well as SSVEPs evoked by a BCI-AO game featuring flashing action images. All patients were shown a video displaying repetitive grasping actions under four conditions. The details are described below and in Figure 1. The experiments were conducted in a randomized order, with intervals of at least 3 days between sessions to avoid potential carry-over effects (Figure 2).

The patients were instructed to perform each experiment while seated in a relaxed position in a comfortable chair without making any voluntary movement. This was confirmed by monitoring muscle activity throughout the experiment. The game implemented in this study included a video showing the movement of the hand and forearm holding a ball, and was individually executed by setting the direction of the arm in the game to the direction of the affected arm. The BCI-AO program was designed to provide rewarding visual feedback, highlighting the enlargement of the muscles in the hand and forearm based on the user's level of attentive watching. The video blinked at a frequency of 15 Hz to allow the classifier to detect the patient's SSVEP components in the EEG. The details are elaborated upon in the following sections.

#### 2.2.1 Experimental conditions

In this study, the settings for the four conditions were determined by combining the PES and BCI-AO as follows and in Figure 1:

(1) In the BCI-AO without PES application (nPES condition), patients were instructed to observe the movement of the left or right (affected) hand and forearm holding the ball on the video screen. Patients received visual feedback whether their attentive watching was assessed by detector. However, they didn't provide PES during the task.
(2) In the BCI-AO with continuous PES application (cPES condition), patients followed the same BCI-AO paradigm as in the nPES condition. The difference was that continuous PES was applied while the video played on the screen, irrespective of whether patient's attentive watching was verified or not.
(3) In the BCI-AO with triggered PES application (tPES condition), the PES was turned on and off synchronously based on the patient's state of attentive watching. This allowed patients to receive visual feedback and synchronized PES simultaneously, with the PES application being dependent on attentive watching.
(4) In the BCI-AO with reverse PES application (rPES condition), the PES was operated in the opposite manner compared to the tPES condition. Specifically, the PES was turned off during periods of attentive watching and turned on during periods of low attentiveness. As a result, the participants were exposed to asynchronous PES, in which PES was applied in the opposite manner to tPES condition.

#### 2.2.2 BCI-AO system

The BCI-AO paradigm was created by presenting a blinking video clip designed to evoke SSVEP patterns in the EEG of the patient while watching it. The video evokes SSVEP and stimulate brain activity in motor-related regions by providing appropriate visual feedback (forearm muscles in the AO video) to encourage users to watch videos of grasping movements according to the patient's attention. EEG data were obtained using DSI-24 with 19 electrodes (Wearable Sensing, San Diego, USA). The electrodes were positioned following the international 10–20 system. Data were collected at a frequency of 300 Hz, and the adequacy of the EEG signal quality was checked before each experiment.

Before each experimental session, a calibration session was carried out to collect data for designing an individual classifier. This session included a total of 15 trials over 3 min. Each trial involved a 6 s video of blinking actions followed by the display of a white cross pin on a black background for an additional 6 s.

The data were organized through a classifier design stage, which consisted of a classifier design and a Common Spatial Filter (CSF) design. CSFs were designed for alpha (8–13 Hz), beta (13–30 Hz), and SSVEP (14.5–15.5 Hz) bands, after which the CSFs were applied in the classifier design phase. The classifier was built using a Support Vector Machine (SVM) algorithm in the conformation of a linear kernel.

After designing of the classifier and CSF, the implementation of BCI-AO became feasible. The processing of EEG signal step for the runtime application consisted of applying band-pass filters and CSF to each feature, and then classifying the state according to each feature using an SVM classifier. We performed AO on patients classified as attentive watching during all conditions and saved the results as EEG epochs with a window size of 1 s segmented at intervals of 0.1 s, allowing the BCI system to offer feedback at intervals of 0.1 s and then the classified state was applied to the PES trigger immediately which could be considered as real-time.

In the runtime application, the BCI-AO measured the attentional ratio, defined as the degree of attentive watching during the running time. The average score was 56% ± 3.123, 58% ± 4.336, 60% ± 4.156, and 57% ± 2.563 during the nPES, cPES, tPES, and rPES conditions, respectively. The attention ratio was computed by expressing the time during which mu suppression was observed as a percentage of the total task time. EEG processing was performed using the OpenViBE software platform ver. 2.3.0 (INRIA, Rennes, France).

#### 2.2.3 Peripheral nerve stimulation

PES was performed using a commercial system (Medel GmbH, Germany) with a single bipolar channel applied to the affected limb to inject a current (with a pulsed square waveform) into the right ulnar nerve. PES was delivered at 120% of the intensity of the sensory thresholds of stationary FDI muscles to the extent that muscle contraction did not occur, and at frequencies of 50 Hz and 1 ms pulse widths while patients were relaxed.

To avoid potential disturbances in the EEG signal by PES, a frequency of 50 Hz was selected for real-time EEG filtering in the BCI-AO program, which operates within the range of 0.1–50 Hz. The EEG signal was then filtered in three different bands (8–13 Hz, 13–30 Hz, and 14.5–15.5 Hz) to extract features for the real-time BCI program.

The stimulus intensity was set at 120% of the sensory threshold, defined as the lowest stimulus detected by the patient. The choice of intensity setting of the stimulus was based on the fact that PES above the motor threshold causes muscle spasms. Therefore, all the patients were able to tolerate this intensity of stimulation without complaining of muscle spasms or pain.

In each condition, the overall intensity of PES administered to the patients was expressed as the PES intensity (mA) for 15 min. The analysis revealed that the PES intensities under the cPES, tPES, and rPES conditions were 3,720, 2,153, and 1,674 mA, respectively.

### 2.3 Assessment

The time points for measuring the latency and amplitude of MEPs and F wave latency are illustrated in Figure 2. MEP parameters were assessed at three intervals: baseline (Pre), immediately after the task training (Post 0), and 20 min after completing each task (Post 20). F-wave latencies were evaluated at baseline (Pre) and immediately after the task training (Post 0). Additionally, we quantified mu suppression in EEG activity during each of the conditions.

#### 2.3.1 Motor evoked potential

Motor induced potential (MEP) was achieved using Magstim 200^2^ (The Magstim Company Limited, Whitland, UK) and D70^2^ coils to measure cortical stimulation. Measurements of MEPs using MagStim were performed by a fully experienced physician in an electrical diagnostic laboratory. The coil orientation was applied to the mid-phase plane at a 45° posterior angle, and the hat featured hotspot points marked with the grid at 1 cm intervals to ensure a consistent stimulation area throughout the examination. The resting motor threshold (rMT) was determined as the minimum stimulation intensity with a MEP amplitude >50 μV recorded five times out of ten. The stimulation intensity was consistently set at 120% of the rMT and maintained throughout the experiment. Twenty MEPs were recorded with a stimulus interval of 5–6 s, and average peak-to-peak amplitude and latency were subsequently computed.

#### 2.3.2 F-wave

To evaluate peripheral nerve excitability, the latency of the F-waves in the FDI muscle was measured by stimulating the ulnar nerves in the wrist using a Medelec Synergy electromyography machine (Natus Neurology Inc. USA) before and after each task. We maintained a temperature of 22°C in the laboratory to exclude temperature-dependent variations in the F response.

#### 2.3.3 EEG analysis

To measure mu suppression during each condition, we extracted mu-band powers from the EEG data and the corresponding resting data, which were filtered between 4 and 30 Hz, applied an artifact subspace reconstruction filter to the filtered EEG data for artifact rejection, and then calculated mu suppression from the power spectrum density of the EEG using the following equation:


10×log10 (mu_power_of_task/mu_power_of_rest).

The left and right electrode positions were then reversed when necessary to match the data from the affected and unaffected sides of the patient.

### 2.4 Sample size

The main goal of this study was to evaluate the impact of four different conditions on corticospinal excitability in stroke patients. Sample size calculations were conducted using G Power 3.1.9.7 (Heinrich Heine University, Dusseldorf, Germany) for Windows. The determination of the sample size for repeated-measures ANOVA was made considering the primary endpoints. We analyzed four sets of conditions to identify differences in the MEP amplitudes between pre vs. post 0 and between pre vs. post 20 in stroke patients. We determined that a sample size of 15 patients was adequate for detecting an effect value of 0.26 (large effect size) (Ward, 2005). Additionally, the correlation among repeated measures of 0.7 was found to have a significance level of 0.05 (both sides) for a power of 90%. Consequently, we decided on a sample size of 15 patients and recruited 17 patients to account for potential dropouts.

### 2.5 Statistical analysis

Data are provided as estimated mean and standard error. Differences between measured values at baseline and after the tasks were calculated. The normality of continuous variables was assessed through the Kolmogorov–Smirnov test and the Shapiro-Wilk test. We applied the linear mixed-effects model to assess the impact of four conditions (nPES, cPES, tPES, and rPES), time (Pre, Post 0, Post 20), and the interaction between condition and time on MEP latencies and amplitudes. We applied the linear mixed model to analyze the impact of four conditions, time (Pre, Post 0) and the interaction between condition and time on the latency of the F wave. A three-way linear mixed model was employed to evaluate the effect of other variables such as age, sex, etiology, Modified Rankin Scale (mRS), Fugl-Meyer Assessment for Upper Extremity (FMA-UE), MMSE, Modified Ashworth Scale (MAS), as well as attentional ratio and intensity of PES during the tasks. When significant *p*-values were observed for variables in the three-way interaction, they were divided based on the median values, and a linear mixed model was employed. A repeated measures analysis of variance (ANOVA) was conducted to evaluate the differences in mu suppression among the four tasks on the C3/C4 and F3/F4 channels of the affected hemisphere in stroke patients. When significant differences were identified, *post-hoc t*-tests were conducted.

The results were defined as statistically significant if the *p*-value was < 0.05, and trends were recognized if the interaction *p*-value was < 0.15. All data were analyzed using IBM SPSS Statistics software (version 26.0; IBM Corp., Armonk, NY, USA), and SAS software (version 9.4; SAS Institute, Cary, NC, USA).

## 3 Results

### 3.1 Baseline characteristics

Twenty-eight patients with hemiplegic stroke were assessed, and all patients underwent a screening test to determine the measurability of MEP parameters. Eleven patients were excluded from the study because clear measurement of MEP parameters was not possible. In total, 17 patients were initially enrolled in the study. However, two patients dropped out due to side effects: one patient withdrew due to headaches during the study and the other due to eye fatigue that arose during the experiments. In the end, MEP results from 15 patients with stroke (10 men and five women) were analyzed. Their detailed clinical and demographic data are shown in Table 1. During EEG analysis, one more patient was excluded because mu-band powers could not be clearly recorded. Thus, the EEG analysis involved a total of 14 patients. The baseline MEP amplitude, MEP latency, and F-wave latency were not significantly different between the four conditions (*F*_3, 84_ = 0.23, *p* = 0.88; F_3, 84_ = 0.34, *p* = 0.79; *F*_3, 42_ = 0.37, *p* = 0.78) (Supplementary Table).

### 3.2 Changes in MEP parameters after experiments

**Figure 3:**
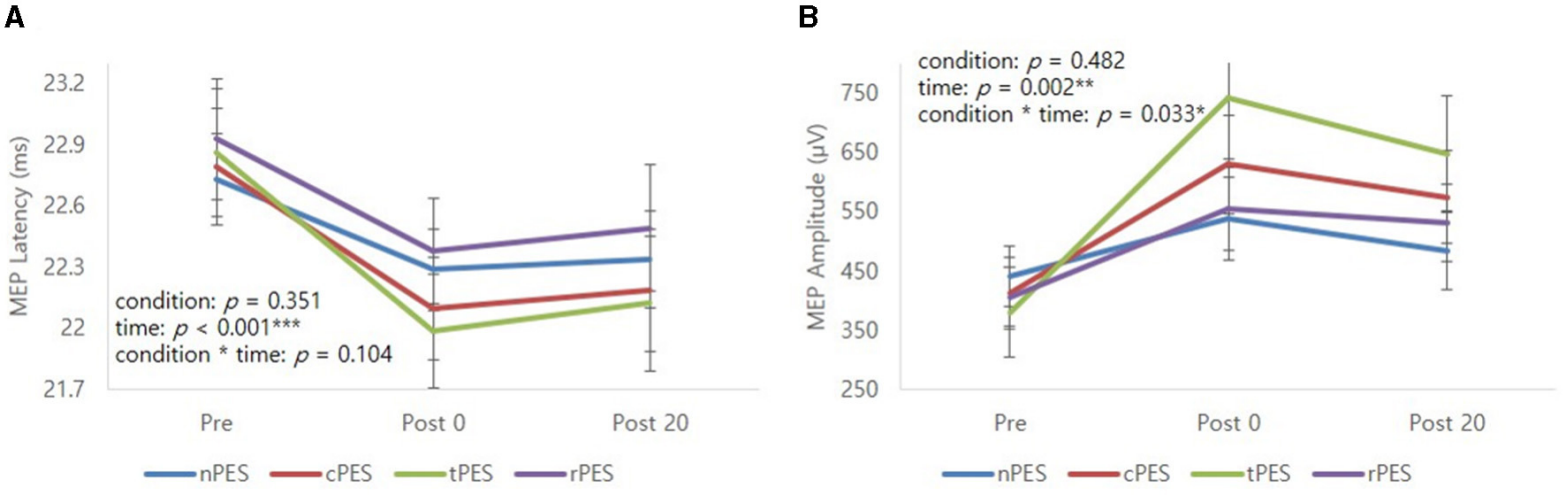
Mean profile graphs showed the significant main effect of time and a trend of condition × time interaction for MEP latency **(A)**, and significant main effect of time and condition × time interaction for MEP amplitude **(B)**. nPES condition (blue line), cPES condition (red line), tPES condition (yellowish green line), rPES condition (purple line). AO, action observation; MEP, motor evoked potential; nPES, BCI-AO without PES application; cPES, BCI-AO with continuous PES application; tPES, BCI-AO with triggered PES application; rPES, BCI-AO with reverse PES application; PES, peripheral electrical stimulation. *p < 0.05; **p < 0.01; ***p < 0.001.

**Figure 4:**
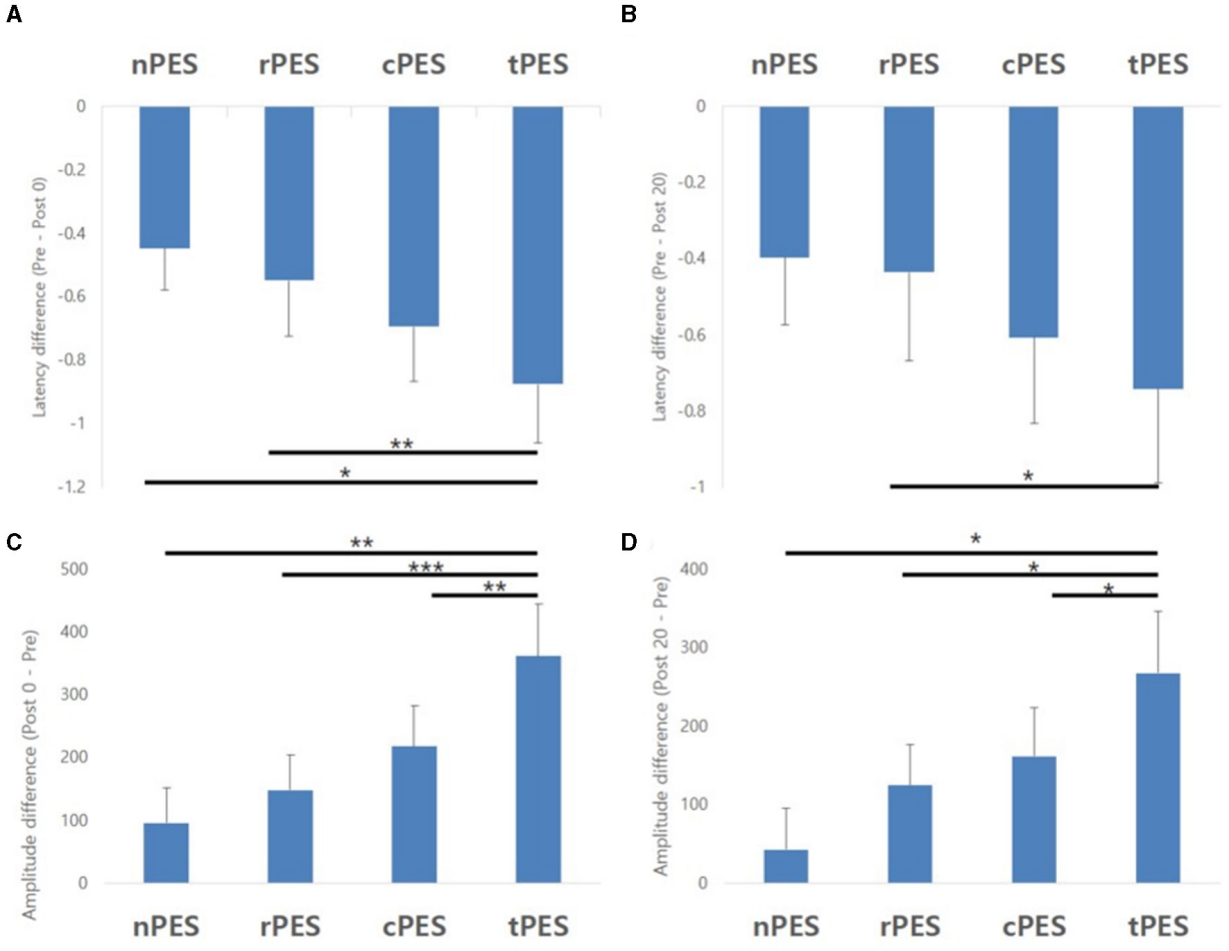
Results of the *post-hoc* analysis for MEP parameters. They showed a significant decrease in MEP latency between tPES vs. rPES condition and tPES vs. nPES condition **(A, B)**. A significant increase in MEP amplitude was observed between tPES and nPES condition, tPES and rPES condition, tPES and cPES condition **(C, D)**; MEP, motor evoked potentials, nPES, BCI-AO without PES application; cPES, BCI-AO with continuous PES application; tPES, BCI-AO with triggered PES application; rPES, BCI-AO with reverse PES application; AO, action observation; PES, peripheral electrical stimulation; BCI-AO, brain–computer interface-action observation. *p < 0.05; **p < 0.01; ***p < 0.001.

Table 2 and Figure 3 show the changes in MEP parameters among the four conditions in patients with stroke. There was a significant main effect of time (Pre, Post 0, Post 20; *F*_2, 28_ = 9.93, *p* = 0.005) and a trend in the interaction between condition and time (*F*_6, 84_ = 1.82, *p* = 0.1044) on MEP latency. However, no significant effect of the condition was observed (*F*_3, 42_ = 1.12, *p* = 0.351). *Post-hoc t*-tests revealed a significant reduction in MEP latency after the nPES, cPES, tPES, and rPES conditions compared to that at baseline (Pre vs. Post 0). These differences in MEP latency persisted after 20 min, with no significant differences observed between Post 0 and Post 20 under any of the four conditions (Figure 3). The *post-hoc t*-tests further revealed a significant decrease in MEP latency between the tPES and rPES conditions (Pre-Post 0: *t* = 2.89, *p* = 0.005; Pre-Post 20: *t* = 2.04, *p* = 0.045), and between the tPES and nPES conditions (Pre-Post 0: *t* = 2.55, *p* = 0.013). However, there were no significant differences in the MEP latency between the nPES and cPES conditions, the nPES and rPES conditions, the cPES and tPES conditions, or the cPES and rPES conditions (Figure 4).

**Table 2:** Changes in MEP parameters among the four conditions.

**Outcome**	**Time**	**nPES condition**	**cPES condition**	**tPES condition**	**rPES condition**
Latency (ms)	Pre	22.735 (0.223)	22.793 (0.287)	22.864 (0.314)	22.930 (0.295)
	Post 0	22.289 (0.197)	22.100 (0.254)	21.990 (0.277)	22.383 (0.260)
	Post 20	22.340 (0.235)	22.188 (0.302)	22.124 (0.329)	22.495 (0.310)
Amplitude (μV)	Pre	441.581 (50.949)	412.843 (60.272)	380.570 (75.567)	407.349 (50.790)
	Post 0	537.802 (69.941)	630.647 (82.738)	742.088 (103.735)	555.911 (69.722)
	Post 20	484.981 (66.107)	574.948 (78.202)	648.695 (98.048)	532.051 (65.900)
F wave (ms)	Pre	26.850 (0.466)	26.730 (0.305)	26.950 (0.376)	26.797 (0.458)
	Post 0	26.797 (0.497)	26.730 (0.326)	26.943 (0.401)	26.747 (0.489)

All values are given as the estimated mean and standard error for each condition and three different time points: pre (before task), post 0 (after task), and post 20 (20 min after task). nPES, BCI-AO without PES application; cPES, BCI-AO with continuous PES application; tPES, BCI-AO with triggered PES application; rPES, BCI-AO with reverse PES application; AO, action observation; PES, peripheral electrical stimulation; BCI-AO, brain–computer interface action observation.

We observed a significant main effect of time (*F*_2, 28_ = 8.26, *p* = 0.002) and an interaction between condition and time (*F*_6, 84_ = 2.43, *p* = 0.033) on the MEP amplitude. *Post-hoc* analysis for time indicated a significant increase in MEP amplitude after the cPES, tPES, and rPES conditions compared to baseline (Pre vs. Post 0), with no significant increase after the nPES condition; however, the increase in MEP amplitude following the cPES, tPES, and rPES conditions remained significant for 20 min (Pre vs. Post 20), and no differences were found between Post 0 and Post 20 in the cPES, tPES, and rPES conditions (Figure 3). The *post-hoc* analysis on the interaction between condition and time revealed a significant increase in the MEP amplitude between the tPES and nPES conditions (Post 0: *t* = 2.83, *p* = 0.006; Post 20: *t* = 2.54, *p* = 0.013), tPES and rPES conditions (Post 0: *t* = 3.57, *p* = 0.001; Post 20: *t* = 2.54, *p* = 0.013), and tPES and cPES conditions (Post 0: *t* = 2.69, *p* = 0.009; Post 20: *t* = 2.10, *p* = 0.039). However, no significant differences were observed between nPES and cPES, nPES and rPES, and cPES and rPES conditions (Figure 4). The estimated mean difference in MEP amplitude after the task was highest between the nPES and tPES and rPES and tPES conditions (Table 3).

**Table 3:** Estimated mean difference and *p*-value from the *post-hoc* analysis of MEP amplitude.

**Condition**	**Pre vs. post 0**		**Pre vs. post 20**		**Post 0 vs. post 20**	
nPES vs. cPES	121.582 (75.359)	0.1104	118.704 (71.287)	0.0996	−2.878 (65.303)	0.965
nPES vs. tPES	265.297 (93.583)	0.0057^**^	224.725 (88.527)	0.013^*^	−40.572 (81.096)	0.6182
nPES vs. rPES	52.341 (55.652)	0.3497	81.301 (52.645)	0.1263	28.960 (48.226)	0.5498
cPES vs. tPES	143.715 (53.380)	0.0086^**^	106.021 (50.496)	0.0388^*^	−37.694 (46.257)	0.4174
cPES vs. rPES	−69.241 (47.390)	0.1477	−37.403 (44.830)	0.4065	31.838 (41.067)	0.4403
tPES vs. rPES	−212.956 (59.733)	0.0006^***^	−143.424 (56.505)	0.013^*^	69.532 (51.762)	0.1828

All values are expressed as estimated mean and standard errors (pre: before task, post 0: after task, post 20:20 min after task). ^*^p < 0.05; ^**^p < 0.01; ^***^p < 0.001. MEP, motor-evoked potentials; nPES, BCI-AO without PES application; cPES, BCI-AO with continuous PES application; tPES, BCI-AO with triggered PES application; rPES, BCI-AO with reverse PES application; AO, action observation; PES, peripheral electrical stimulation; BCI-AO, brain–computer interface action observation.

Time, condition, and the interaction between them did not exhibit any significant effects on the F-wave latency (see Supplementary Figure).

### 3.3 Effects of other variables in MEP parameter

The Mini-Mental State Examination (MMSE) showed a significant main effect on MEP latency (*F*_6, 143_ = 2.70, *p* = 0.016). In patients with MMSE scores under 28, there was a significant effect of the interaction between condition and time on MEP latency (*F*_6, 24_ = 11.86, *p* < 0.001). However, this was not seen in those with MMSE scores over 29 (*F*_6, 54_ = 1.72, *p* = 0.135). There was no effect of sex (*F*_6, 78_ = 1.17, *p* = 0.333), age (*F*_6, 143_ = 1.44, *p* = 0.202), etiology (*F*_6, 78_ = 1.52, *p* = 0.183), mRS (*F*_12, 72_ = 0.82, *p* = 0.629), FMA-UE (*F*_6, 143_ = 0.32, *p* = 0.924), MAS (*F*_6, 78_ = 1.77, *p* = 0.117), the attentive ratio (*F*_6, 142_ = 0.57, *p* = 0.755) or intensity of PES (*F*_4, 103_ = 0.60, *p* = 0.662) on MEP latency.

Moreover, there was a significant main effect of age (*F*_6, 143_ = 3.53, *p* = 0.003) on MEP amplitude. There was a significant effect of interaction between condition and time in patients aged ≥65 years (*F*_6, 24_ = 3.60, *p* = 0.006), but not in those aged < 64 years (*F*_6, 36_ = 1.61, *p* = 0.172) on MEP amplitude. There was no effect of sex (*F*_6, 78_ = 1.05, *p* = 0.995), etiology (*F*_6, 78_ = 2.16, *p* = 0.056), mRS (*F*_12, 72_ = 1.82, *p* = 0.061), FMA-UE (*F*_6, 143_ = 1.40, *p* = 0.219), MMSE (*F*_6, 143_ = 1.80, *p* = 0.103), MAS (*F*_6, 78_ = 1.29, *p* = 0.273), attentional ratio (*F*_6, 142_ = 0.96, *p* = 0.452), or PES intensity (*F*_4, 103_ = 1.31, *p* = 0.270) on MEP amplitude.

### 3.4 Changes in mu suppression during the experiments

**Figure 5:**
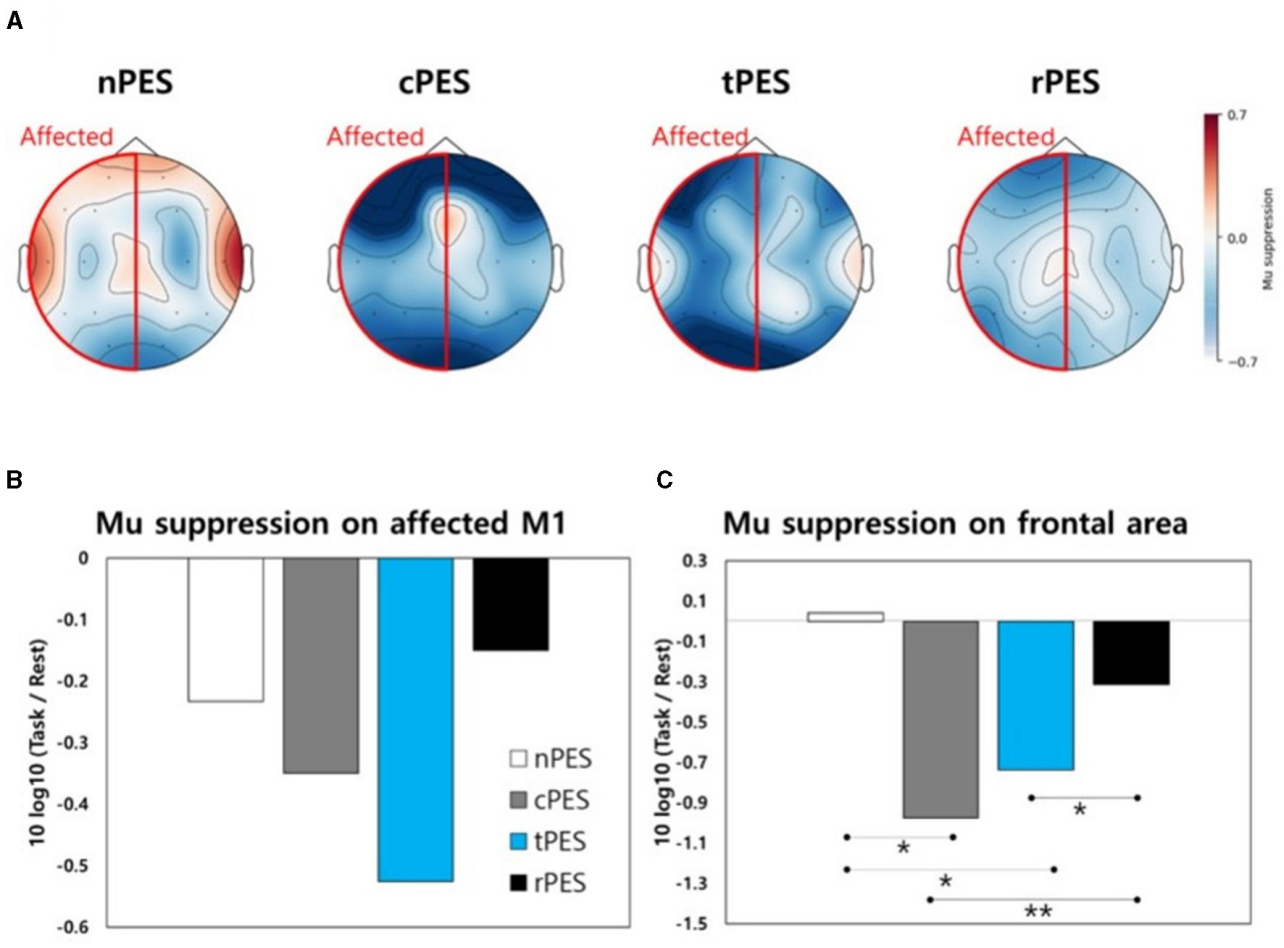
**(A)** Topographical representation of mu suppression in EEG during the four conditions. Mu suppression is observed in the motor area **(B)** and frontal area **(C)** of the affected hemisphere during the four conditions. nPES, BCI-AO without PES application; cPES, BCI-AO with continuous PES application; tPES, BCI-AO with triggered PES application; rPES, BCI-AO with reverse PES application; AO, action observation; PES, peripheral electrical stimulation; BCI-AO, brain–computer interface-action observation. *p < 0.05; **p < 0.01.

Figure 5A shows a topographical representation of mu suppression. Mu suppression of the affected hemisphere was strongest in the cPES and tPES conditions compared to the nPES and rPES conditions. In addition, when we assessed mu suppression in the motor cortex of the affected hemisphere (C3 or C4), there was no statistically significant difference between conditions (Figure 5B). Regarding mu suppression in the affected frontal area (F3 or F4), significant main effects were observed in the four conditions (*F*_3, 39_ = 6.272, *p* = 0.001), with the most pronounced mu suppression observed in the cPES condition. The *post-hoc* analysis showed significantly stronger activation in the cPES and tPES conditions compared to the nPES and rPES conditions (cPES vs. nPES: *p* = 0.013; tPES vs. nPES: *p* = 0.018; cPES vs. rPES: *p* = 0.003; tPES vs. rPES: *p* = 0.037, Figure 5C).

### 3.5 Side effects during the experiments

After each experimental session, all patients were checked for the presence of any side effects, such as neck pain, dizziness, or headache. Two patients dropped out from the study due to side effects. One patient reported a headache, and the other reported eye fatigue. Most of the other patients completed the study without experiencing any discomfort.

## 4 Discussion

This study demonstrated that different PES pairings during BCI-AO task training can modulate corticospinal plasticity after a stroke. We found evidence that the corticospinal excitability in the affected hemisphere after a stroke was higher when PES was applied synchronously with AO training, dependent on a highly attentive brain state. Conversely, the effects of corticospinal activation were not significant when PES was applied continuously or in the reverse manner. Hence, the synchronous application of PES with attentional state AO training using BCI distinctly influenced the modulation of corticospinal plasticity, supporting our initial hypothesis. This paradigm continued to promote cortical plasticity for a short period after it was stopped.

It is well-known that AO promotes motor re-learning in patients with stroke by activating the MNS and motor cortex (Zhang et al., 2018). However, our study did not reveal a significant increase in MEP parameters after AO alone. This finding is consistent with that of a previous study (Kim et al., 2022) that suggested that the corticospinal plasticity induced by AO was insufficient in patients with chronic stroke. PES, known to stimulate the motor cortex through afferent stimulation during AO-induced activation, likely contributes to the induction of long-term potentiation (LTP)-like plasticity in a manner similar to overt movement execution (Bisio et al., 2017).

The paradigm that integrated a BCI video game with AO induced more significant mu suppression in the motor cortical area than AO alone in patients with stroke (Choi et al., 2019). Our BCI-AO paradigm is innovative and different from other BCI systems (Kim et al., 2016). In particular, it offers real-time rewarding visual feedback to aid patients to focus on the training and enhance their rehabilitation. Reward feedback strategies promote motor skill consolidation through motor network activation during training (Widmer et al., 2016). Motivation is an essential factor for active participation, and active patient engagement is crucial for the success of rehabilitation training in patients with stroke (Kusec et al., 2019). Thus, the BCI-AO paradigm can facilitate motor skill recovery by maintaining patient motivation.

In a previous study, the BCI-AO with PES paradigm was superior to AO with PES and AO alone in promoting corticospinal plasticity in both healthy individuals and in patients with stroke (Kim et al., 2022). Moreover, the MNS can be successfully activated during electrical stimulation synchronized with AO using functional Near-Infrared Spectroscopy (Cui et al., 2023). Therefore, our finding of a significant increase in corticospinal excitability and central cortical activation in the EEG analysis following BCI-AO with the PES task is consistent with previous research. The synergistic application of neurorehabilitation therapies, each operating via distinct mechanisms, has the potential to enhance neural plasticity in patients with stroke (Takeuchi and Izumi, 2015). Thus, in this study, activation of the NMS induced by AO, activation of reward-learning attentional networks, and the peripheral nervous system may have enhanced corticospinal plasticity through associative plasticity beyond the motor cortex.

Furthermore, we investigated whether the timing of the BCI-defined stimulation and the pairing of highly attentive brain states with PES application could maximize corticospinal excitability. Importantly, our findings revealed that different PES pairings during BCI-AO training had distinct effects. This indicates that synchronous PES application during attentive watching was assisted by the BCI rather than by continuous PES application, reinforcing corticospinal plasticity. Conversely, there was a detrimental effect on corticospinal activation resulting from the inconsistency between the state of attentiveness of the brain and the application of PES. Thus, the rPES vs. nPES conditions showed no significant difference in corticospinal excitability. Corticospinal excitability was not affected by the overall state of attention (attentional ratio) or the PES intensity during each task. Although the overall intensity and duration of PES during the task was greater in the cPES condition than in the tPES condition, the increase in MEP amplitude was only significant in the tPES condition. These findings suggest that synchronous PES is crucial to a user's highly attentive brain state. In our novel BCI-AO paradigm dependent on brain state, the cortical state of attentiveness and PES have a stable and synergistic effect in the activation of the corticospinal system. Importantly, this paradigm sustained corticospinal activation for 20 min after task completion.

It has been suggested that simultaneous combinations of neurotechnologies for motor recovery are not always effective in patients with stroke (Coscia et al., 2019). Homeostatic plasticity might diminish the synergistic effects of simultaneous combinations (Takeuchi and Izumi, 2015). To maximize neural plasticity, closed-loop stimulation paradigms using BCI, which synchronize the stimulation of postsynaptic neurons with activity in presynaptic neurons, aim to engage more synergistic patterns of neuronal activity in the associative process (Mrachacz-Kersting et al., 2016). Thus, the plasticity effects induced by this paradigm are likely to translate more directly into functional gains compared to those from an open-loop paradigm (Ethier et al., 2015). Recent studies strongly support the hypothesis that timing-dependent, cue-based electrical stimulation enhances corticospinal neuroplasticity (Fu et al., 2021; Niazi et al., 2022). The results of an earlier study showed that PES application paired with EEG-defined movement intention enhanced MEPs (Niazi et al., 2012). More importantly, the change in corticospinal excitability was only observed when the PES was paired with cortical states corresponding to movement initiation, but not at the onset of muscle activation (Fu et al., 2021). When PES was paired with visual cue-based BCI interventions, there was a significantly increased MEP amplitude and exercise performance compared to PES application without visual cues (Niazi et al., 2022). Similarly, employing consistent coupling of repetitive TMS with the high-excitability state defined by the mu rhythm resulted in LTP in corticospinal excitability, whereas no significant change was observed when the coupling was to the low-excitability state or to a random mu rhythm phase (Zrenner et al., 2018).

In the present study, the latency of the ulnar nerve F-waves at the wrist showed no significant changes across the four conditions. This suggests that there were no notable changes at the peripheral or spinal levels after each task. This outcome aligns with those of a prior investigation wherein the application of PES alone did not induce modifications in F-wave responses (Ridding et al., 2000; Kaelin-Lang et al., 2002). Therefore, it is likely that the observed changes in MEP parameters after the task mainly originated from the cortex rather than from spinal or peripheral sources.

Whereas, participants aged ≥ 65 years showed significant differences in MEP amplitudes across the four conditions, those aged < 64 years did not. This finding implies that corticospinal activation in older patients with stroke may be task-specific and more effective when stimulation dependent on the brain state is provided. Activity-dependent plasticity and LTP-like plasticity after motor learning are reduced in older individuals (Zimerman et al., 2013; Ghasemian-Shirvan et al., 2020). A previous study reported EEG findings indicating attention deficits during visual memory tasks in older people (Teng et al., 2018). In addition to the effect of aging, stroke induces structural and functional changes in the brain, resulting in decreased motor cortical activation. In a previous study, the integration of BCI-AO with the PES paradigm led to a shift in the brain activation pattern toward the central brain area (Lim et al., 2023). This paradigm induced more extensive utilization of the frontal and motor areas in stroke patients than the AO alone or the AO+PES paradigms. Therefore, older patients with stroke appear to require a training strategy that is dependent on the brain state to enhance attentiveness and cortical facilitation for motor recovery. Furthermore, patients with MMSE scores of < 28 exhibited significant differences in MEP latency across the four conditions, unlike those with MEP scores of >29. Although we exclusively recruited patients with relatively good cognitive function (MMSE score >24), the results suggest that individuals with lower cognitive abilities may respond more effectively to stimulation that is dependent on brain state.

Cortical activation at the central site, as indicated by mu suppression, displayed some notable patterns during the four tasks according to the topographical analysis (Figure 5A). In the frontal area, the cPES and tPES conditions elicited stronger activation than the nPES and rPES conditions. However, these findings were not statistically significant in motor cortex. There are two potential explanations for the lack of statistically significant results for the motor cortex in the EEG analysis. First, the sample size may have been insufficient because it was calculated based on a previous TMS study following stroke (Mrachacz-Kersting et al., 2019). Secondly, the distinct spatial and temporal resolutions of the TMS and EEG methods may have influenced the results (Lapenta et al., 2018). In agreement with the TMS results, the rPES condition, in which PES was applied in the opposite manner to tPES, revealed mu suppression patterns in the frontal area that were relatively similar to those observed in the absence of PES application (Figure 5C).

This study has some limitations. First, we only investigated short-term changes in corticospinal plasticity after training. Corticospinal activation lasted for 20 min after task completion, confirming that the present paradigm promoted cortical plasticity for a short period, even after it was stopped. In earlier studies, the repetitive application of BCI to detect the cortical state associated to motor intention in conjunction with electrical stimulation induced improvement in upper-extremity motor function after stroke (Biasiucci et al., 2018; Mrachacz-Kersting et al., 2019). Hence, further investigation of the impact of our novel paradigm on functional recovery after stroke is required. Second, we included relatively few patients with mild to moderate stroke impairment. As mentioned earlier, our paradigm has the advantage of being applicable even to severely paralyzed stroke patients, because it is not dependent on motor activity. Unfortunately, this study excluded patients with severe motor weakness, in whom MEPs in the affected hand muscles could not be elicited. Therefore, further research is necessary to investigate patients with severe paralysis using alternative brain imaging techniques. Third, we administered PES below the sensory threshold, at a frequency of 50 Hz. The intensity and frequency of stimulation can influence corticospinal excitability (Pitcher et al., 2003; Chipchase et al., 2011; Saito et al., 2013). Considering that the optimal PES settings may vary between different patients, future research is required to determine how to maximize the therapeutic effect in each individual case. Forth, this study employed an exploratory approach to analyze the results, considering the small sample size, so that there was a limitation in that no corrections were made for multiple comparisons.

## 5 Conclusion

This study demonstrated that different pairings of PES application during BCI-AO influenced the task-dependent corticospinal plasticity after stroke. We provided new insight indicating that corticospinal excitability in the affected hemisphere increased when PES was applied synchronously with an AO task using BCI during a state of high attention, compared to continuous or asynchronous PES application. This paradigm promoted corticospinal plasticity for up to 20 min after task completion. Importantly, this effect was more evident in patients over 65 years. The ideal neurorehabilitation treatment for elderly patients with stroke is likely to be multimodal, involving not only the incorporation of effective feedback to create a highly motivational environment but also the optimization of techniques that are dependent on the brain state for sufficient task-induced neural plasticity. This study extended these results by integrating BCI technologies dependent on the attentional state with other conventional rehabilitation training methods.

## Data availability statement

The original contributions presented in the study are included in the article/Supplementary material, further inquiries can be directed to the corresponding authors.

## Ethics statement

The studies involving humans were approved by Institutional review board of Nowon Eulji Medical Center/Eulji University (2022-07-012-001). The studies were conducted in accordance with the local legislation and institutional requirements. The participants provided their written informed consent to participate in this study.

## Author contributions

CJ: Investigation, Writing—original draft, Methodology, Resources, Project administration. HL: Investigation, Methodology, Software, Writing—original draft, Resources, Visualization. JL: Investigation, Methodology, Writing—original draft, Resources. HL: Formal analysis, Writing—review & editing, Data curation. JK: Conceptualization, Data curation, Validation, Writing—review & editing, Formal analysis. YK: Conceptualization, Funding acquisition, Supervision, Validation, Writing—review & editing.
