# Supplementary material for: Attentional state-synchronous peripheral electrical stimulation during action observation induced distinct modulation of corticospinal plasticity after stroke
Source: Front Neurosci. 2024 Mar 28;18:1373589. doi: 10.3389/fnins.2024.1373589 (PMC11007104; doi:10.3389/fnins.2024.1373589)
Supplement: Supplementary file 1 [file Table_1.DOCX]

Supplementary Material

# Supplementary Figure

**Supplementary Figure.** **Changes in spinal and peripheral excitability.** Mean profile graphs of spinal and peripheral excitability parameters. They did not show any significant main effect of time, condition, and condition * time interaction for spinal and peripheral excitability parameters; nPES condition (blue bar), cPES condition (orange bar), tPES condition (yellowish bar), rPES condition (gray bar) (**p* < 0.05; ***p* < 0.01; ****p* < 0.001); nPES, BCI AO without PES application; cPES, BCI AO with continuous PES application; tPES, BCI AO with triggered PES application; rPES, BCI AO with reverse PES application; AO, action observation; PES, peripheral electrical stimulation; BCI AO, brain–computer interface-action observation.

# Supplementary Table: The baseline difference of MEP parameter of both group

| Outcome(pre) | nPES condition | cPES condition | tPES condition | rPES condition | *P*-value |
| --- | --- | --- | --- | --- | --- |
| Latency (ms) | 22.735 (0.223) | 22.793 (0.287) | 22.864 (0.314) | 22.930 (0.295) | 0.3506 |
| Amplitude (µV) | 441.581 (50.949) | 412.843 (60.272) | 380.570 (75.567) | 407.349 (50.790) | 0.4821 |
| F wave (ms) | 26.850 (0.466) | 26.730 (0.305) | 26.950 (0.376) | 26.797 (0.458) | 0.7935 |

All values are expressed as estimated mean differences. In addition to the estimated mean difference, standard errors were calculated (pre- and pre-task). nPES, BCI AO without PES; cPES, BCI AO with continuous PES; tPES, BCI AO with triggered PES; rPES, BCI AO with reverse PES; AO, action observation; PES, peripheral electrical stimulation; BCI AO, brain–computer interface observation. **p* < 0.05; ***p* < 0.01; ****p* < 0.001.
